# Impact of Perceived Supportive Learning Environment on Mathematical Achievement: The Mediating Roles of Autonomous Self-Regulation and Creative Thinking

**DOI:** 10.3389/fpsyg.2021.781594

**Published:** 2022-01-06

**Authors:** Weihua Niu, Li Cheng, Dana Duan, Qingyang Zhang

**Affiliations:** ^1^Department of Psychology, Pace University, New York, NY, United States; ^2^Faculty of Education, Beijing Normal University, Beijing, China; ^3^Developmental and Educational Research Center for Children’s Creativity, Beijing Normal University, Beijing, China

**Keywords:** perceived supportive learning environment (PSLE), mathematics achievement, autonomous self-regulation, creative thinking, meditation analysis

## Abstract

A total of 1,281 Chinese students in grades 3–6 participated in a study that examined the relationships among student-perceived supportive learning environment (PSLE), mathematical achievement, autonomous self-regulation, and creative thinking. The results demonstrated that student PSLE is positively associated with autonomous self-regulation, creative thinking, and mathematical achievement. In addition, the study also demonstrated that the influence of PSLE on students’ mathematical achievements could be mediated through autonomous self-regulation and creative thinking, respectively. The results shed light on the effectiveness of a supportive learning environment on educational and psychological outcomes in Chinese mathematical classrooms.

## Introduction

Mathematics is one of the most critical educational subject areas in modern society. A student’s mathematical ability in elementary school not only influences their mathematical achievement in later years but can also predict their future educational outcomes and career successes (Cross et al., 2009). In addition, while mathematical ability in elementary school establishes the foundation for all other technical fields, math skills have become increasingly important in the digital era in which algorithmic language and artificial intelligence are used in everyday life (Schleicher, 2019). Therefore, cultivating learners’ mathematical ability to adapt to this new mode of communication is a critical topic in today’s world.

How can a teacher help elementary school students improve their mathematical achievement? Studies have shown that the following factors played important roles in children’s learning outcomes. These factors include teachers’ pedagogical belief (Staub and Stern, 2002), clear and structured instruction, an emphasis on building up complex skills (such as deductive and critical thinking) in conjunction with skill acquisition (Griffin, 2004; Cameron et al., 2005; Connor et al., 2007), and classroom climate a teacher can provide to students (Crosnoe et al., 2010; Wang et al., 2020).

Among all these factors, classroom climate provides the most critical microsystems directly affecting student learning process and outcomes (Fraser, 2012, 2019; Ehun-Shik, 2019). Classroom climate is a complex multidimensional construct, including at least three essential components: teacher-student interaction, instructional support, and social-emotional support (Wang et al., 2020). A growing interest in recent literature focuses specifically on supportive learning environments a teacher-created in classroom settings. A supportive learning environment or SLE can make students feel included, valued, and empowered. Unlike a traditional classroom setting, an SLE focuses more on the relationships among all participating individuals in the class. It encourages students to interact with the teacher, freely share their perspectives and engage in future-orientated learning (Ju-Sen and Chaoyun, 2014; Khine et al., 2020).

There is sufficient evidence to support the positive relationship between SLE and academic achievement; nevertheless, the mechanism behind the connection has not been fully established. This study contributes to the literature by examining two indirect pathways from SLE and academic achievement in Chinese elementary mathematics classrooms. The two pathways look at the mediating roles of students’ autonomous self-regulated motivation and creative thinking in the relationship between students’ perceived supportive learning environment (PSLE) and mathematical achievement.

## Literature Review

### Educational Outcome of Supportive Learning Environment

Many studies have examined the relationship between SLE and educational outcomes such as academic engagement and achievement.

Scott et al. (2007) studied the impact of teachers’ positive behavior support in the classroom on elementary students’ problem behaviors and academic achievement through case studies. They found that problem behaviors decrease and academic performance increase with help from teachers through positive reinforcement.

Observing 1,238 preschoolers over 101 classes, Schenke et al. (2017) found that children’s achievements benefited from teachers with a supportive attitude and developmentally appropriate expectations. This effect was stronger among African American children than non-African American children.

Using a field experiment, Liao and Wang (2015) examined the role of the supportive learning environment in Taiwanese university students’ learning outcomes by comparing two different classrooms; one served as a control class in which lectures are the primary mode of instruction. The other served as experimental class downplaying authoritative and competitive learning environments to let students feel empowered and supported to get involved in dual- or multi-person interactions with their instructors and peers. They found that students from the experimental class received better academic achievement in English than those from the control class, suggesting that a supportive learning environment was conducive to academic learning.

Through a survey design, Kearney et al. (2016) examined the relationship between students’ perceived teacher support in the classroom and elementary school students’ academic engagement. They found that a supportive learning environment is conducive to elementary school students’ learning outcomes. Also, *via* a survey, Baek and Choi (2002) studied the relationship between classroom environment and academic achievement among Korean high school students. They found that students’ perceived positive classroom environment significantly predicted students’ academic achievement. Similarly, Sakiz et al. (2012) found that students’ perceived teacher support positively predicted middle school students’ educational outcomes. In addition, teacher leadership styles could indirectly impact the academic achievement of students in middle school (Leithwood and Jantzi, 2000).

Several systematic literature reviews and meta-analysis studies further solicited the relationship between SLE and educational outcomes. For example, Núñez and León (2015) conducted a systematic literature review and concluded that many desirable outcomes, such as academic performance, creative thinking, and engagement in school, benefit from classrooms where teachers support autonomy. Korpershoek et al. (2016) conducted a meta-analysis of 54 studies published between 2003 and 2013 to examine the effects of classroom management on students’ academic, behavioral, social-emotional, and motivational outcomes in primary education, and the results revealed small yet significant effects on all outcome variables, except for motivational factors. Using a systematic review and meta-analysis of 61 studies published between 2000 and 2016 examining the relationship between supportive classroom climate and children’s learning, Wang et al. (2020) found that supportive classroom climate had small-to-medium positive links with social competence motivation and engagement, and academic achievement. Moreover, they found negative associations between socio-emotional distress and externalizing behaviors.

In summary, there is a wealth of evidence to support that SLE can lead to many desirable educational outcomes among students in all age groups, from preschoolers to university students worldwide.

### Supportive Learning Environment, Autonomous Self-Regulation, and Academic Achievement

What are some possible pathways to explain how SLE affects educational outcomes? One mechanism is through students’ motivation. In other words, how students perceive the learning environment might affect their motivation to learn and then subsequently affect their learning. According to self-determination theory, individuals’ motivation can be either autonomous or controlled. Autonomous motivation can be in the form of intrinsic (driven by their interest and enjoyment of the activity) or identified regulation (driven by personal significances of their behavior). Controlled motivation can be in the form of introjected regulation (driven partially but not entirely, internalized, to seek self-worth or avoid shame and guilt and external regulation (caused by externally pressuring demands such as attaining a controlling reward or avoiding criticism from significant others (Deci and Ryan, 1985; Ryan and Connell, 1989; Ryan and Deci, 2000). The degree of autonomous self-regulation is used to represent how individuals act for their interests and take control of the process of their learning (Ryan and Deci, 2000).

Many studies have examined the relationship between SLE, motivation, and academic success but primarily focused on reviewing two variables at the time (e.g., Ryan and Patrick, 2001; Zuo and Tan, 2002; Sheng and Zhang, 2005; Zhang and Ning, 2010). For example, Zhang and Ning (2010) found a significant positive correlation between learning motivation and achievement in mathematics among Chinese elementary school students. Ryan and Patrick (2001) also found that teachers’ support and views on promoting interaction and mutual respect were positively associated with student motivation to learn and engage in mathematics learning.

Some recent studies solicited the mediating role of motivation in the relationship between learning environment and students’ academic success. For example, Hughes et al. (2012) surveyed 690 elementary students at risk for academic failure included in the study. They found that students’ motivation mediated the effects of student-reported teacher-student relationship quality (conflict and warmth) on reading and math achievement. Froiland et al. (2016) further demonstrated that high school students’ autonomous self-regulation mediated perceived teacher support of autonomy and their mathematics performance. Investigating 512 junior high school students, Liu et al. (2021) found that autonomous motivation significantly mediated the relationship between perceived teacher support and creative self-efficacy.

In other words, it is plausible that autonomous self-regulation mediates between SLE and academic achievement. In other words, the goal of SLE is to promote students’ sense of autonomy in learning, which is a driving force for their academic success.

### Supportive Learning Environment, Creative Thinking, and Mathematic Achievement

Another possible pathway is through student creativity. Creativity is defined as a person’s ability to create something deemed both original and appropriate by experts in a given domain (Amabile et al., 1996; Sternberg, 1999; Beghetto and Kaufman, 2009; Simonton, 2012). Creativity often involves divergent and convergent thinking processes (Guilford, 1967), and the learning environment, particularly the classroom environment, has a crucial influence on the development of students’ creativity (Richardson and Mishra, 2018).

Most studies supported that a perceived creative learning environment could positively impact students’ creativity (e.g., de Souza Fleith, 2000; Besançon and Lubart, 2008; Hu, 2016; Sun et al., 2019; Ahn and Cho, 2021). For example, investigating 470 seventh and eighth-graders in China, Hu (2016) found that perceived creative classroom environment (i.e., teacher-student relationship, students’ cohesiveness, involvement, cooperation, teaching method, and equity) positively impacted students’ creative thinking. Sun et al. (2019) also found that perceived teacher support positively predicts convergent thinking and insight thinking, with creative self-efficacy partially mediating between perceived teacher support and convergent thinking.

Other studies have found a positive association between creativity and academic performance. For example, Hansenne and Legrand (2012) found a positive association between creativity and academic performances in French and mathematics in a French elementary school. Bano et al. (2014) found a significant positive association between creativity and academic achievement in many subject areas among high school students in Pakistan. The coefficient was higher for math than it was for any of the other subject areas.

However, not all studies have shown that creativity positively predicts academic achievement. For example, Gralewski and Karwowski (2012) found no correlation between student creativity and math scores among high school students in Poland. It is important to note that the latter two studies were conducted in high schools, in which the academic pressures are more elevated. Notably, the positive correlation between creativity and academic performance seems more substantial and consistent in elementary school than in high school (Gajda et al., 2017). Regardless, further studies must be conducted to verify the relationship between the two.

Few studies have explored the mediating role of creative thinking in the relationship between learning environment and academic achievement. Cheng et al. (2019) examined the relationship among students’ perceived creative classroom environment, creative thinking, and academic achievement in Chinese language and literacy (CLL) among children with higher general intelligence. The researchers concluded that divergent thinking mediated certain variables of student perceived creative classroom environment and academic achievement in CLL. More specifically, when teachers create a classroom environment that encourages student interaction, students’ divergent thinking is promoted, and as a consequence, their academic performance improves.

### The Current Study

Previous studies have shown that an SLE can positively influence academic achievement and may also indirectly affect self-regulated motivation or creative thinking pathways. However, to our knowledge, there lacks empirical evidence that connects all four variables, namely, supportive learning environment, self-regulated motivation, creative thinking, and academic achievement, together in a single study.

In this study, we measured the SLE through students’ self-report on their perceptions of the supportive learning environment in school. More specifically, we propose that students’ PSLE positively impacts their autonomous self-regulation and creative thinking, subsequently promoting their mathematics achievement. To control the influence of grade and general ability, we measured students’ IQ and used standardized scores at grade levels in mathematics to represent mathematics achievement.

Based on the studies discussed in previous sections, we propose three hypotheses: First, PSLE would positively correlate with students’ mathematic achievement, automatic self-regulation, and creative thinking. Second, autonomous self-regulation mediates the relationship between PSLE on mathematic achievement. Lastly, creative thinking mediates the influence of PSLE on mathematics achievement.

## Materials and Methods

### Participants

The participants were 1,281 third to sixth graders (644 females) from 20 mixed-gender classes in a mega-primary school in North China. The student characteristics may have changed as a result of enrollment. Several years ago, students were primarily from the surrounding rural communities, and with urbanization, the school has gradually attracted students from the adjacent urban neighborhoods. Students in the current 5th and 6th grade enrolled in school in their early years, and students in 3th and 4th students enrolled later, so students’ family backgrounds may differ. Among the participants, three hundred eighty-five students (194 females) were in the 3rd grade, three hundred fifty-one (193 females) were in the 4th grade, three hundred seventy-three (180 females) were in the 5th grade, and one hundred seventy-two (77 females) were in the 6th grade.

### Procedure and Measures

Before the start of the study, researchers contacted the school and explained the purpose of the study. The local school examined and approved the research and sent a letter to all parents requesting their consent. Only those students with parental consent participated in this study. The study was conducted during a school day near the end of the semester. Students completed the survey in three blocks of time. Each block consisted of the following: creativity tasks, intelligence tests, and other measurements presented in random order, with breaks in between. All of these measurements have been used extensively in previous research and deemed age-appropriate for elementary school children. The participants were told that they could withdraw from the study or skip any questions at any time without penalty. All research procedures were approved by the Research Ethics Review Board of the authors’ institution (the ethical approval code was BNU202106100014).

The measurements included the following.

#### Tony Non-verbal Intelligence Test (TONI-2)

The TONI-2, which was initially developed by Brown, Sherbenou, and Johnson (TONI) in 1982 and then revised as TONI-2 in 1990, is used to test non-verbal abstract/figure problem-solving abilities in eight areas, including shape, position, direction, rotation, contiguity, shading, size, and movement for individuals of ages five through eighty-five.

Zhang et al. (2003) revised the test and created a version to be applied on China’s mainland and then constructed the norm. The overall internal consistency of the TONI-2 is.89, and the split-half reliability is.88. It was also determined to have a higher validity based on Raven’s standard progressive matrices and student achievement. In the study, participants’ average IQ in each grade ranges from 101 to 108, which is a representative sample at average intellectual levels.

#### Creative Classroom Environment Scale – Perceived Supportive Learning Environment

We used the Creative Classroom Environment Scale developed by Cheng et al. (2019) to evaluate students’ perceptions of the factors supporting the development of creativity in the classroom. We used this measurement as the variable for SLE in our study. The scale has 31 items and includes five subscales: teacher leadership, student communication, student relationship, teacher support, and class participation.

Teacher leadership refers to teachers’ classroom guidance to facilitate student learning (e.g., Teachers can capture student attention). Student communication relates to students’ in-class discussion and exchange (e.g., expressing my understanding of problems to other students). Student relationship refers to the mutual support, assistance, and friendship among students (e.g., I can quickly establish friendships with the students in the class). Teacher support refers to providing appropriate relationships and assistance for students who need it (e.g., When I encounter difficulties in learning, the teacher helps me). Class participation refers to the involvement in-class activities to express their understanding of problems (e.g., In-class discussion, I can express my opinions). All items are rated on a 5-point Likert scale ranging from 1 (*almost never*) to 5 (*almost always*).

The internal consistency of the entire scale was 0.93, with the internal consistency for each subscale ranging from 0.77 to 0.86. The first-order model of confirmatory factor analysis showed that the scale had an acceptable model fit, χ2(424, *N* = 1,281) = 1469.935, *RMSEA* = 0.044, *GFI* = 0.929, *CFI* = 0.926, and *RMR* = 0.068, and the second-model of confirmatory factor analysis also fit well, χ2(429, *N* = 1,281) = 1666.674, *RMSEA* = 0.047, *GFI* = 0.918, *CFI* = 0.913, and *RMR* = 0.080). Because the five subscales were highly correlated with each other and can all be extracted to a high-order factor, the average score of five subscales was used to represent the perceived supportive learning environment.

#### Academic Self-Regulation Questionnaire

We used the Academic Self-Regulation Questionnaire (SRQ-A) to measure students’ degree of autonomous self-regulation in learning. Ryan and Connell developed the SRQ-A based on self-determination theory (Ryan and Connell, 1989). This questionnaire includes four subscales to measure motivation on a continuum from external to internal control. The four subscales include (1) nine items regarding external motivation, i.e., gain rewards or avoid punishment (e.g., I do my homework, so teachers don’t yell at me); (2) nine items regarding introjected motivation, i.e., gain self-esteem or avoid guilt (e.g., I do my homework because I’ll be ashamed of myself if it isn’t completed get done); (3) seven items regarding identified motivation, i.e., the motivation that people understand and recognize the value and accept it (e.g., I do my homework because it is important to me); and (4) seven items regarding intrinsic motivation, i.e., gain self-satisfaction, (e.g., I do my homework because I enjoy it). The SRQ-A uses a 4-point Likert scale ranging from 1 (*not at all true*) to 4 (*very true*).

In this study, the internal consistency of the entire scale was 0.85, with the internal consistency of each subscale ranging from 0.72 to 0.85. Based on the manual, a relative autonomy index (RAI) score is calculated to represent the degree of autonomous self-regulation using the following formula: RAI = 2 × intrinsic motivation + identified motivation – introjected motivation – 2 × external motivation. We will consider the autonomous self-regulation as a whole rather than each of the four subscales. The higher the index, the higher the degree of autonomy.

#### Evaluation Potential of Creativity

We used an adapted version of the EPoC instrument developed by Lubart et al. (2011) to measure creativity. The EPoC instrument consists of eight tasks, four convergent-integrative (CI) tasks, and four divergent-exploratory (DE) tasks, which address two content domains, namely, verbal-literary (V) and graphic (G). Furthermore, the following four dimensions of creativity were measured: (1) divergent verbal (DV), (2) divergent graphics (DG), (3) integrated verbal (IV), and (4) integrated graphics (IG). In measuring DV, the participants were given the beginning of a story and were then required to write as many possible endings to the story as they could. In measuring DG, the participants were given an abstract or concrete graphic and were then required to paint as many pictures as possible based on the given figure. In measuring IV, the participants were given three-story elements and were then required to write an original story according to the elements. Finally, to measure IG creativity, the participants were given eight abstract or concrete graphics and were then required to select at least four of them from which they were to create a novel picture.

One rater scored the fluency of two divergent tasks, i.e., one DV and one DG, by counting the number of answers written by the participants. Two trained graduate students who were blinded to the study procedure served as judges and were provided with a rating rubric for scoring the originality of the two integrated tasks (one IV and one IG). Raters were asked to rate all responses for the two tasks separately using a 7-point Likert scale, with 1 denoting the lowest score and seven denoting the highest score possible for originality. The interrater reliability scores (α) on the two originality scores for the creativity tasks were 0.81 and 0.99. The construct validity test indicated that the measurement is valid, with an overall model fit where [χ2(1, *N* = 1,281) = 0.912, *RMSEA* = 0.000, *GFI* = 1.000, *CFI* = 1.000, and *RMR* = 0.00].

There are significant positive correlations among the four sub-scores of creative thinking (*p* < 0.01), so we first converted the raw scores of all four creativity dimensions, DG, DV, IG, and IV, into Z scores. The average Z scores of DG and DV were used to represent a person’s fluency, and the average Z scores of IG and IV were used to describe originality. The final score for creative thinking was calculated by averaging the two Z scores on fluency and originality.

In addition to completing these measurements, all students completed a demographic sheet asking about their gender, age, and ethnicity. Moreover, with the consent of the two participating schools, we also obtained the students’ mathematics scores on their mid-term and final examinations in the spring semester of 2019. The raw scores were then converted into Z scores to represent participants’ math achievement scores for further analysis.

## Results

### Descriptive Statistics

Table 1 shows the general means, standard deviations, and correlation coefficients of the perceived supportive learning environment (PLSE), autonomous self-regulation (RAI), creative thinking (CT), and mathematics achievement (MA). The results suggest that PLSE was significantly positively associated with autonomous self-regulation, creative thinking, and mathematics achievement. Both autonomous self-regulation and creative thinking were significantly positively associated with mathematics achievement. Our first hypothesis was confirmed.

**TABLE 1:** Descriptive statistics and first-degree correlations of main variables (*n* = 1,281).

	Mean	*SD*	1	2	3	4	5	6	7
PLSE (1)	3.35	0.68	1	0.356**	0.150**	0.255**	0.106**	−0.018	−0.011
RAI (2)	−3.73	20.1		1	0.117**	0.187**	0.087**	−0.060*	0.094*
CT (3)	50.21	5.8			1	0.296**	0.156**	0.243**	0.079**
MA (4)	0.03	0.84				1	0.420**	−0.016	0.011
IQ (5)	106.42	11.03					1	−0.211**	0.008
Grade (6)	–	–						1	0.041
Gender (7)	–	–							1

*MA, mathematics achievement (standardized scores at grade level); CT, creative thinking; RAI, relative autonomy index; PSLE, perceived supportive learning environment.*

*p < 0.05, a significant correlation between the variables at the 0.05 level (two-tailed).

**p < 0.01, a significant correlation between the variables at the 0.01 level (two-tailed).

### Mediation Analysis

**FIGURE 1:**
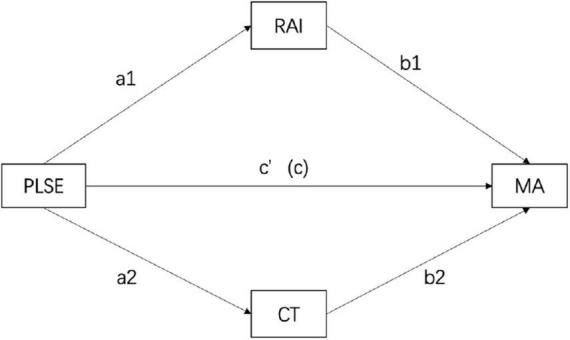
Mediation paths. MA, mathematic achievement (standardized scores at grade level); CT, creative thinking; RAI, relative autonomy index; PSLE, perceived supportive learning environment.

We performed the mediation analyses to evaluate the mediating roles of autonomous self-regulation and creative thinking in the relationship between PSLE and mathematical achievement, controlling grade, gender, and IQ (see Figure 1). Path *a1*, from PSLE to autonomous self-regulation, was significantly positive (95% CI: 8.9387 11.9751), the path *b1*, from autonomous self-regulation to mathematics achievement, was significantly positive (95% CI:0.0011.0054). Multiplying the two effects resulted in the indirect effect (*a1* × *b1*). The indirect effect from PLSE through autonomous self-regulation to mathematics achievement was significantly positive (95% CI:0.0127.0567), which supported our hypothesis 2. Moreover, path *a2*, PLSE to creative thinking, was significantly positive (95% CI:0.7104 1.5909), the path *b2*, from creative thinking, was significantly positive (95% CI:0.0221.0368); multiplying the two effects resulted in the indirect effect (*a2* × *b2*). The indirect effect from PLSE through autonomous self-regulation to mathematics achievement was significantly positive (95% CI:0.0190.0507), which also supported our hypothesis 2.

Table 2 shows the mediating effect of autonomous self-regulation and creative thinking between a perceived supportive learning environment and mathematics achievement. The total effect from PLSE to mathematics achievement (*c*) was 0.2643, and the total indirect effect value was 0.0681 (*c-c′* = a1 *b1 + a2 *b2). Specifically, there are two indirect effects: path 1- PSLE→RAI→MA (a1 *b1 = 0.0342), path 2 – PSLE→CT→MA (a2 *b2 = 0.0339). The ratios of the two indirect effects to the total effect are 12.9 and 12.8% for paths 1 and 2, respectively. The 95% confidence interval of the above indirect effects does not contain zero, indicating that two indirect effects reach a significant level.

**TABLE 2:** Direct and indirect effects of paths.

	Effects	Boot SE	Boot LLCI	Boot ULCI
Total effect	0.2643	0.0308	0.2039	0.3248
Total direct effect	0.1962	0.0322	0.1330	0.2594
Total indirect effect	0.0681	0.0136	0.0415	0.0948
PSLE→RAI→MA	0.0342	0.0110	0.0133	0.0564
PSLE→CT→MA	0.0339	0.0081	0.0186	0.0508

*MA, mathematic achievement (standardized scores at grade level); CT, creative thinking; RAI, relative autonomy index; PSLE, perceived supportive learning environment.*

## Discussion

This study proposed three hypotheses: one direct pathway and two mediating pathways from PSLE to mathematics achievement. First, our results confirmed the direct path from PSLE to mathematics achievement. The results demonstrated that when students actively participate in class activities and interact with teachers and peers, they are more likely to engage in deeper mathematical learning and thereby experience enhanced mathematics achievement. This finding is consistent with findings from previous research (e.g., Baek and Choi, 2002; Kosko, 2012; Sakiz et al., 2012; Wong et al., 2018).

We also proposed two indirect pathways that PSLE could have on mathematical achievement, and the results confirmed both paths. In other ways, in addition to a direct influence of PSLE on mathematical achievement, PSLE can also stimulate one’s autonomous self-regulation and promote student creative thinking, which subsequently supports mathematical achievement. These results are consistent with previous studies in which students’ mathematical achievement could be enhanced through the mediating role of intrinsic motivation (León et al., 2015). Similarly, our results are consistent with previous findings that creative thinking could mediate between students’ perceived classroom environment and academic achievement (Cheng et al., 2019).

Our study further examined the mediating effects of autonomous self-regulation and creative thinking in the relationship between PSLE and mathematics achievement. In other words, a supportive environment can simultaneously stimulate students’ autonomous self-regulation and promote creative thinking; as a consequence, both would further increase their mathematics achievement. This result supports Niu et al. (2017) ‘s findings. Chinese mathematics teachers often spend a significant amount of time and energy creating an SLE that stimulates students’ interests in mathematical learning and encourages them to engage in divergent thinking in classroom activities.

To some extent, this result explained the Asian paradox in mathematical learning. The class sizes in many Asian countries, especially China, are typically twice or even three times greater than the class sizes of many Western classrooms. However, students from China and other East Asian societies consistently score in the top percentiles on many international mathematical achievement tests such as the PISA (Cheng and Hsu, 2016). Many scholars have attributed these high performances to the significant amount of time engaged in the learning process in the mathematics classroom and the amount of time devoted to homework in Asian countries (Stevenson et al., 1986; Stigler and Perry, 1988). Others have also attributed this phenomenon to the cultural values of many eastern Asian countries, which emphasize the importance of academic success (eg., Ralston et al., 1992; Niu, 2007; Lee, 2008; Zhou et al., 2012; Niu et al., 2017). It is essential to point out that results from PISA only represent selective cities from each country and may not reflect the actual academic achievement of all students from each country. We also recognize that students have various needs and backgrounds that could affect their learning outcomes.

Despite the abovementioned reasons, a critical reason that many Western observers overlook is that autonomic self-regulation and divergent thinking are essential educational goals in Chinese math classrooms. Moreover, teachers devote a significant amount of time and energy to enhancing their skills to create a supportive teaching environment that promotes students’ interest in learning mathematics and divergent thinking in mathematics classrooms. All of these efforts eventually lead to positive outcomes in math learning among Asian students.

Our findings support existing literature showing that a supportive learning environment, especially with a greater emphasis on teacher-student and student-student relationships, is critical for students’ learning outcomes (Thompson and Wheeler, 2008). Our study also demonstrated that a supportive environment could effectively promote students’ automatic self-regulation and improve their creative thinking. This finding provides further evidence that teachers alone can play a critical role by influencing students’ academic performance and other essential skills such as creativity and self-regulated learning, which can long-term impact students’ lives beyond academics.

## Limitations

This study examined how PSLE can effectively promote students’ learning outcomes through autonomous self-regulation and creative thinking mediators. However, a cross-sectional study is not sufficient to thoroughly examine mediating effect. Therefore, further studies should include a longitudinal design and an intervention program to determine how the two mediators impact academic performance. Moreover, as this study confirmed the mediating model in mathematics, future research can further examine this new model in other subject areas, such as language, literature, and STEM mediators.

## Conclusion and Implications

In conclusion, findings from this study demonstrated that students’ perceived supportive learning environment (PSLE) in mathematical classrooms is positively associated with their mathematical performance. Moreover, there appear to be two indirect pathways between PSLE and mathematics achievement; that is, PSLE can simultaneously trigger students’ automatic learning, facilitate creative thinking, and enhance their mathematical performance.

As mentioned previously, decades of research have shown that a natural, supportive learning environment created by a teacher should allow students to feel included, empowered, and valued in the classroom. These experiences can promote students’ autonomy and self-regulation, which often lead to optimal learning outcomes. Our findings further demonstrated the critical roles of autonomous self-regulation and creativity in learning mathematics. In other words, intrinsic motivation and creative thinking contribute to a student’s success in mathematics.

Our study has many real-life implications, especially for Asian educators. Although Asian students often obtained higher scores in mathematics achievement tests than other students, their creativity is hindered by their super high stake test-driven educational system (Niu, 2007). It may take a long time and a concerted effort to change the educational testing system in Asian societies. Therefore, it may be more manageable for educators (i.e., teachers and parents) to create a supportive learning environment in classrooms and at home. SLE can effectively diminish the negative effect of test-driven education on creativity and protect Asian students’ automatic self-regulated learning, promoting student creativity, and overall learning outcome, such as continued success in mathematical achievement.

## Data Availability Statement

The raw data supporting the conclusions of this article will be made available by the authors, without undue reservation.

## Ethics Statement

The studies involving human participants were reviewed and approved by the Beijing Normal University. Written informed consent to participate in this study was provided by the participants’ legal guardian/next of kin.

## Author Contributions

LC organized the procedure of the research. DD and QZ performed the statistical analysis and wrote the first draft of the manuscript. WN and LC revised the manuscript. All authors contributed to the study and read and approved the submitted version.

## Conflict of Interest

The authors declare that the research was conducted in the absence of any commercial or financial relationships that could be construed as a potential conflict of interest.

## Publisher’s Note

All claims expressed in this article are solely those of the authors and do not necessarily represent those of their affiliated organizations, or those of the publisher, the editors and the reviewers. Any product that may be evaluated in this article, or claim that may be made by its manufacturer, is not guaranteed or endorsed by the publisher.
